# Nutrition-Related Adverse Outcomes in Endurance Sports Competitions: A Review of Incidence and Practical Recommendations

**DOI:** 10.3390/ijerph17114082

**Published:** 2020-06-08

**Authors:** José Miguel Martínez-Sanz, Ana Fernández Nuñez, Isabel Sospedra, Alejandro Martínez-Rodríguez, Raúl Domínguez, José Antonio González-Jurado, Antonio J. Sánchez-Oliver

**Affiliations:** 1 Departamento de Enfermería, Grupo de Investigación en Alimentación y Nutrición (ALINUT), Facultad de Ciencias de la Salud, Universidad de Alicante, 03690 Alicante, Spain; josemiguel.ms@ua.es (J.M.M.-S.); isospedra@ua.es (I.S.); 2 Facultad de Ciencias de la Salud, Universidad de Alicante, 03690 Alicante, Spain; anafndez@gmail.com; 3 Analytical Chemistry, Nutrition and Food Sciences Department, Sciences Faculty, University of Alicante, 03690 Alicante, Spain; amartinezrodriguez@ua.es; 4 Facultad de Ciencias de la Salud de la Universidad Isabel I, 09003 Burgos, Spain; raul.dominguez@ui1.es; 5 Facultad del Deporte, Universidad Pablo Olavide de Sevilla, 41013 Sevilla, Spain; 6 Departamento de Motricidad Humana y Rendimiento Deportivo, Universidad de Sevilla, 41004 Sevilla, Spain; sanchezoliver@us.es

**Keywords:** heat stroke, hyponatremia, dehydration, hypothermia, gastrointestinal diseases, sport, athletic performance

## Abstract

During the last few years, the numbers of competitors in endurance and ultra-endurance sports modalities have increased significantly. This type of competition is an extreme challenge for athletes. Therefore, they have an increased the risk of developing medical and nutritional problems. The aim of the work is to estimate the incidence of nutrition-related adverse outcomes in endurance and ultra-endurance sports, considering the variables that influence them. A critical review was carried out based on the PubMed database, by means of a search strategy based on keywords separated by Boolean connectors. For all the results obtained in a period from 2008 to 2019, a series of inclusion/exclusion criteria was applied to select only the studies that fitted the objective of the present study. Results and discussion: Of the 871 publications identified, 33 met the inclusion criteria. The adverse outcomes found included exercise-associated hyponatremia (EAH), heat stroke by exertion (EHS), gastrointestinal (GI) problems, dehydration, and hypothermia; the provision of misinformation to athletes about nutrient intake and hydration during competition was identified as the main cause. Conclusions: The main adverse outcomes in endurance and ultra-endurance sports modalities are EAH, GI inconveniences, and EHS. These problems can affect the performance and health status of the athlete during and post-competition. Several nutritional guidelines have been suggested that can prevent these adverse outcomes, and it is essential to individualize and adjust the nutritional intake and hydration status according to the characteristics of each competition.

## 1. Introduction

Endurance sport modalities requires competitors to complete a set distance or amount of work in the shortest time possible (time trial) or a maximal amount of work in a fixed amount of time [1] with the predominance of an energy metabolism dependent on oxidative processes [2,3]. In this sense the more important performance variables in endurance sport modalities is the maximal aerobic power, lactate threshold and economy [4] and the duration of the competition are higher than 5 min [1]. Within these modalities, there are ultra-endurance sports, those competitions with a duration of more than 6 h [5]. In recent years there has been a notable and widespread increase in the number of ultra-endurance events (triathlons, marathons, cycling competitions, mountain races, etc.), in which athletes test their physical and mental abilities to the limit [5].

The ultra-endurance competitions pose extreme challenges for athletes, since during them they may be under extreme conditions—for example, due to changes in altitude or extreme temperatures - that increase physical demands and nutritional requirements, as well as psychological conditions that should be regulate to facilitate their performance [5,6]. These competitions require the athletes to focus on different factors such as the distance of the competition, the profile of the tests, or the different stages or aspects related to the environment (for example, temperature, humidity, and altitude). Therefore, proper training, individualized nutritional preparation, and adequate equipment are essential to successfully complete the event [7]. Thus, athletes that are poorly prepared for these types of competitions have an increased risk of injuries and illnesses [8]. Among these problems and diseases to which athletes are exposed, those related to nutrition, and hydration must be highlighted [9]. These adverse outcomes refers to all the effects that can occur to athletes during a competition or training that are related to their nutrition and that affects the athlete’s health.

Dehydration (DH) is the problem of greatest concern to date, being caused by inadequate fluid intake and/or a significant loss of body fluid through sweat [10]. However, it should be noted that in some environments overhydration is almost as frequent, being a key stimulus for the development of symptomatic hyponatremia associated with exercise (EAH) [11], which in some endurance and ultra-endurance competitions has affected as many as 30% of the participants [12]. The appearance of EAH can be prevented and treated if it is detected quickly and appropriate measures are taken, thus avoiding the complications and side effects that, in extreme cases, can lead to death [13].

The weather conditions constitute a very important factor in endurance and ultra-endurance sports, and may lead to problems for athletes during the competition [14]. During prolonged effort in a hot condition, exercise hyperthermia cause an important challenge at thermoregulatory level [15]. Therefore, the thermic energy associated to the mechanical work caused by muscle contractions increase body temperature and considering that a higher environmental temperature than skin temperature difficult dissipate the thermic energy produced, exercise in hot conditions can provoke stress heat stroke (EHS) [16], an adverse outcome with important consequences, being a potentially dangerous condition for the athletes and one of the first three causes of sudden death in sports [17]. At the opposite, another potentially dangerous weather condition is the cold, together with competitions in a low-temperature aquatic environment, since athletes are exposed to frostbite and hypothermia (HT) [18]. Nevertheless, these situations can be minimized by nutrition. Therefore, the dehydration diminish the ability to dissipate body heat and a correct fluid reposition during exercise could diminish a possible EHS associated to exercise [19]. Likewise, even in cold weather the active body still sweats and needs fluids, and warm fluids or at room temperature can help to protect against hypothermia [20].

Gastrointestinal (GI) problems are also very common in endurance athletes, and it can inhibit sport performance and/or recovery. In general, studies suggest that between 30 and 70% of athletes experience gastrointestinal problems during competition [21]. Most gastrointestinal symptoms during exercise are mild and do not carry any health risks, but hemorrhagic gastritis or intestinal ischemia can be serious medical problems [22]. The most common complaints of the upper gastrointestinal tract include heartburn, nausea, abdominal cramps, vomiting, epigastric pain, and diarrhea. Each of these symptoms may be present in multiple disease states at different levels of severity [23].

An adequate nutritional strategy, according to the characteristics of the athlete and the events he/she participates in, will help to reduce the problems described above, in turn allowing the athlete to successfully complete the events [10]. Thus, the aim of this study is to carry out a critical review to estimate the incidence of nutrition-related adverse outcomes during competition of endurance and ultra-endurance sports.

## 2. Materials and Methods

The work comprised a descriptive study, involving a critical review, to answer the research question, “what is the incidence of the different nutrition-related adverse outcomes that occur in endurance/ultra-endurance competitions and sports?” A structured search was performed in the PubMed database. To find the largest number of available articles related to the research question, the words of the search strategy were established considering: 1) The descriptors of the Medical Subjects Headings (MeSH); 2) other terms described in MeSH as “entry terms”, which include the terminology prior to the setting-up of the MeSH register; and 3) the terms [tiab] or [Title/Abstract] attached to the “entry terms” or MeSH, in singular or plural, which allow the localization of these terms in the title and summary of the articles. The words and the search strategy were in Appendix A.

The articles selected for the literature review met the following inclusion criteria:

- Human studies related to adverse outcomes that occur in endurance and ultra-endurance athletes during competitions.
- Type of study: cohort-study, case studies or clinical trials.
- Published in English or Spanish.
- Articles published from 2008 to 2019.

Dates were limited to offer a most recent and updated information according to the scientific advances in sports nutrition field, because in the lasts 10 years the number of endurance and ultra-endurance competition events has increased substantially and in parallel, nutritional recommendations for sports people have undergone significant changes. Articles in review format, non-human studies, and studies that were not related to athletes or that analyzed sports that did not belong to the endurance or ultra-endurance modalities were excluded. The screening of the articles was carried out independently by two researchers, reviewing the titles and summary, accessing the full text in case of doubt, and agreeing on the inclusion or exclusion of the articles.

The article selection protocol for this study was composed of the following variables:

- Study: authors and year of publication
- Objective: purpose for which the study was carried out.
- Sample: number of subjects trained for the activities, sports discipline, and sex.
- Sports competition: type of competition where adverse outcomes detected, place and year of occurrence.
- Adverse outcomes: type of adverse effect found.
  - Incidence: number or % of cases of adverse outcomes suffered by athletes during the competition.
  - Causes: established after the investigation, triggers of said problems suffered during the competition
- Conclusions: obtained from the research and reached by the authors themselves.

## 3. Results

**Figure 1:**
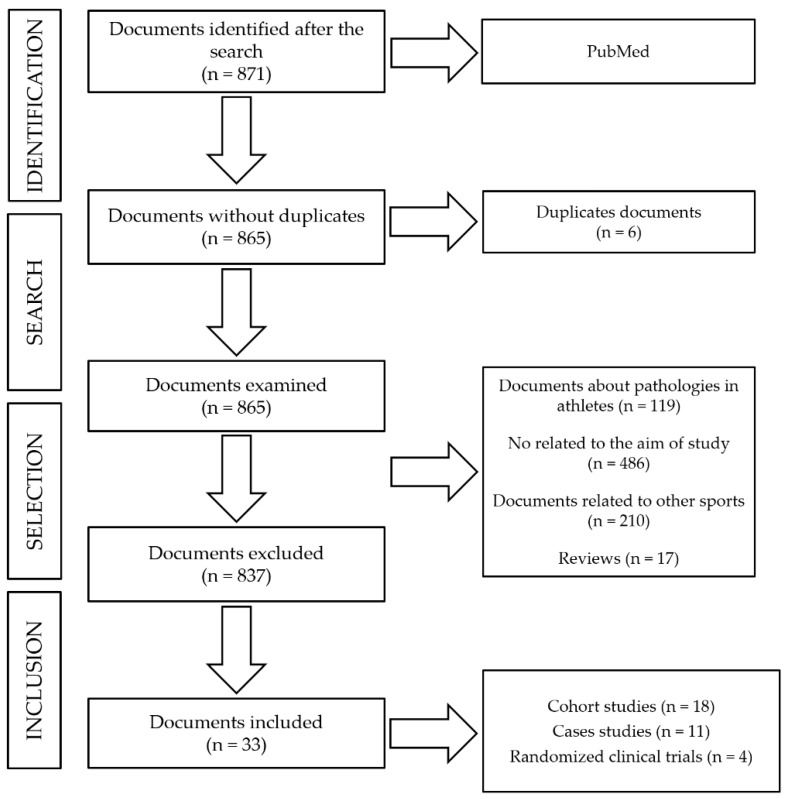
Flow diagram showing the process used to select the studies.

The number of publications identified was 871. After eliminate duplicate articles (*n* = 6), 865 articles were identified for this review, of which only 33 (18 cohort-study articles, 11 case studies, and 4 randomized clinical trials) remained after applying thee exclusion criteria (see Figure 1).

Table 1, Table 2 and Table 3 describe the variables used to analyze the adverse outcomes that arose during competitions and were reported in the studies used in the review. In this way, Table 1 groups the cohort studies, Table 2 shows the randomized clinical trials, and Table 3 the studies based on case studies.

The sample composed of the different works included in the review amounted to a total of 23,344 athletes. As for the country in which the competitions analyzed took place, the different studies included the United States (*n* = 12), followed by the United Kingdom (*n* = 5), France (*n* = 4), Brazil (*n* = 2), the Czech Republic (*n* = 2), Germany (*n* = 1), Australia (*n* = 1), The Netherlands (*n* = 1), Ireland (*n* = 1), Norway (*n* = 1), Portugal (*n* = 1), South Africa (*n* = 1), and Switzerland (*n* = 1).

The sporting competitions analyzed in the different studies were: ultra-marathon (*n* = 8), marathon (*n* = 8), cycling (*n* = 6), Olympic distance triathlon (*n* = 3), hiking (*n* = 3), combined ultra-endurance races (*n* = 2), obstacle courses (*n* = 2), swimming (*n* = 1), and half-marathon (*n* = 1).

Among the adverse outcomes observed in the different studies were GI diseases or alterations (*n* = 7), EHS (*n* = 7), HT (*n* = 3), and alterations related to the hydration status of the athletes: EAH (*n* = 13) and DH (*n* = 5). Among the GI alterations we find nausea, vomiting, diarrhea, stomach/abdominal pain, cramps, and GI hemorrhages.

The incidence of the problems studied was calculated in cohorts studies where total population was described (Table 4). In cohorts studies the data ranged from 6.4% (HT) to 20.4% (DH). The most common adverse events related to weather conditions were DH, which appeared in 392 of 1918 of the athletes studied, followed by EAH with an incidence of 10.3% (290 cases of 2829), GI with 9.4% (1302 cases of 13,852), and HT with 6.4% (13 cases of 202). In clinical trial studies the data of cases ranged from 6.0% (EAH) to 33.3% (DH). Therefore, DH appeared in 10 of 30 of the athletes studied, GI with 5 cases of 30 (16.7%), and EAH with 14 cases of 233 (6.0%). No cases of HT were detected in clinical trial studies, and no cases of EHS were detected in cohorts and clinical trial studies. EAH is one of the most studied adverse outcomes among all the articles included. However, not all the cases were severe; from all the cases reported, only 6% were classified as severe, 8% as moderate, 86% were mild or not clinically significant and for one case no information about its severity was reported.

The authors of the included studies generally concluded by providing recommendations to reduce the likelihood of athletes suffering from the problems described, thus improving their performance, and prioritizing above all an adequate state of health.

## 4. Discussion

**Figure 2:**
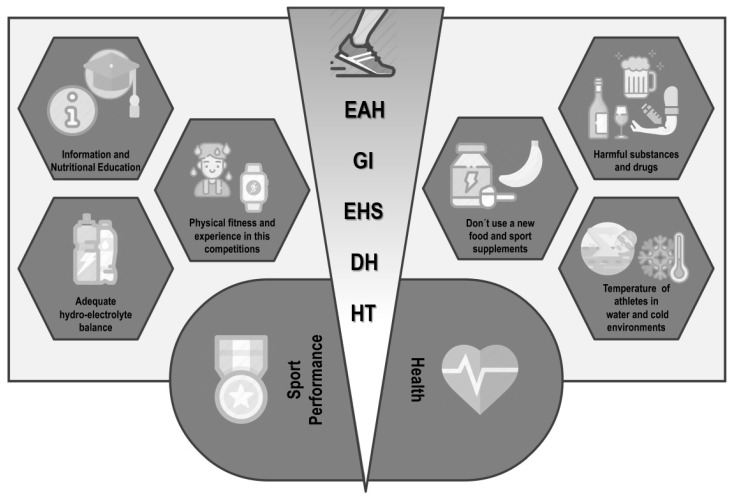
Nutrition-related adverse outcomes in endurance and ultra-endurance sports: key aspects to be considered. EAH: exercise-associated hyponatremia; GI: gastrointestinal problems; EHS: heat stroke by exertion; DH: dehydration; HT: hypothermia.

The main adverse outcomes in competitions of endurance and ultra- endurance are DH, followed by EAH and GI alterations or diseases. The analysis of the results obtained showed that the causes that contribute to the development of some of these risks are of multifactorial origin, although some cause-effect relationships have been established. The inadequate intake of liquid influences the development of DH, as well as an excessive intake can lead to an EAH. Inadequate guidelines on the nutritional needs of each athlete, added to the introduction of food or supplements that the athlete has not previously consumed, can cause discomfort and GI diseases. In addition, if acclimatization appropriate to the conditions in which the competition takes place is not performed, this increases the risk of suffering from EHS and HT (Figure 2).

### 4.1. Exercise-Associated Hyponatremia (EAH) and Dehydration (DH)

EAH is a disorder of fluids and electrolytes that has been widely described in marathon runners [32] and competitors in other endurance and ultra-endurance events such as: cycling races of 24 h or ≥150 km [28,40,43], foot races of 24 h or ≥100 km [28,31], Ironman triathlons [13,32], or open-water swimming events [43]. It seemed to be a rare problem when it was first described in the scientific literature in 1985 [32]; however, the current increase in the incidence of EAH should create alarm among athletes, since it has been confirmed as the cause of at least fourteen deaths, which reaffirms the potential severity of this condition [53].

Studies suggest that ultra-endurance athletes competing in events that exceed 24 h, such as Ultraman, Titan Desert, or Sables Marathon participants, are at a higher risk of developing EAH compared to participants in shorter endurance tests, as could be the case of the marathon [32]. This is confirmed by the results obtained in different investigations, in which incidence rates of 6–18% were reported for Ironman [13,32], 6–11.5% in mountain biking events [28,43], and 15–30% in ultra- endurance running races [34,36], so the duration of the competition is a risk factor for EAH development in endurance and ultra-endurance sport modalities.

For the proper management of EAH in athletes, it is important to make an early diagnosis. However, the signs and symptoms of EAH are nonspecific and can be superimposed on or confused with those of other diseases such as EHS, GI symptoms, and hypoglycemia, which are also frequent in these types of event. Special attention should be paid to the intake of large volumes of fluid, nausea, transient confusion, or exhaustion, as they can alert us to the development of these problems [12,47].

The risk factors that have been identified regarding the development of EAH include: female sex, alcohol consumption, excess of fluid replacement, weight gain during exercise, low body weight, slow running performance and inexperience in endurance events [12]. Another problem that is related to EAH is DH in athletes—which is sometimes followed by EAH, due to excessive hypo-osmotic fluid replacement. According to the results considered in this review, EAH can be also related to the environmental conditions that occur at the time of the competition, since at high temperature or humidity a greater loss of sodium can occur, in sweat [31]. Thirst should provide adequate stimulus for preventing excess dehydration and contribute to reduce the risk of EAH [53]. The incidence of DH may also reflect limited opportunities for the ingestion of fluid during competition and/or a lack of knowledge regarding the nutritional requirements of and fluid consumption by athletes [26].

Since the water needs of athletes tend to differ depending on the individual characteristics and the type or intensity of the exercise in which they participate, individualized fluid replacement strategies are necessary. The American College of Sports Medicine (ACSM) recommends that fluid intake during exercise should limit body weight loss to <2% [54]. This view has been criticized on numerous occasions, as it may be inappropriate for athletes who begin the competition in a severe state of DH [26]. Therefore, and in accordance with the results obtained, the nutritional advice given to endurance and ultra-endurance athletes must include, in addition to adequate personalized and contextualized nutritional information, appropriate advice on hydration that takes into account the requirements of both the individual and the competition. This should result in the prevention of adverse outcomes and an improved performance of the athletes [55].

Both the participants and the organizers of the competitions should be in possession of sufficient information and resources to help reduce the incidence of EAH and DH, ensuring above all the health and safety of all those who are encouraged to participate in endurance and ultra-endurance events.

### 4.2. Gastrointestinal (GI) Problems during Physical Exercise

A high incidence of GI disorders during physical exercise can be observed among long-distance runners [30], duathletes and triathletes [24], cyclists [33,37], and athletes who participate in other sports modalities of long duration [5,25]. The GI problems can be separated into GI symptoms of the upper body—such as gastroesophageal reflux, nausea, vomiting, belching, stomach pain, and swelling—and lower GI symptoms such as abdominal cramps, flatulence, intestinal bleeding, urgency to defecate, and diarrhea [24,26,28,32,54,56]. Some of these GI problems are serious and affect the performance of athletes [23]. In addition, these problems may necessitate the rescue of athletes in mountains; 12.8% of the rescues of mountain athletes in the province of Aragon, Spain, are for these reasons [56].

The occurrence of GI problems during competitions is a common problem for endurance and ultra-endurance runners, who are almost twice as likely to suffer them as athletes of other sports modalities, such as cyclists, swimmers, and other elite athletes. Between 30 and 90% of such athletes may suffer one or more GI problem(s) [23,33] that affect(s) their performance and, therefore, may determine whether or not the athletes finish the competition [23].

The results found highlight diarrhea as the GI disease that affects the highest number of endurance athletes, representing 16.4–59% of the total number of registered cases of GI problems [24,25,30,37,57]. Despite the limited results published in the literature, it has been found that an intake of complex carbohydrates during exercise—instead of consuming only glucose—or the optimal combination of glucose and fructose can prevent the occurrence of this problem. A sufficient amount of carbohydrates should be ingested to ensure the recovery of glycogen stores; this may vary between 5 and 12 g/kg body weight/day depending on the athlete and the physical activity performed [5,58]. If this is combined with a reduced dietary intake of fermentable oligosaccharides, disaccharides, and monosaccharides, the incidence of GI problems is lower [59].

A gluten-free diet has been mentioned as a way of improving the physical condition of athletes; but, since there is no apparent impact on intestinal damage, there is no evidence to recommend this diet for the prevention of GI diseases in non-celiac athletes [60]. Finally, and as a recommendation, it is not advisable to introduce new foods in the diet of athletes, on both the day of the competition and the previous day, since the effects that can be triggered in their bodies are unknown, possibly compromising both their health and their performance [23].

### 4.3. Stress Heat Stroke (EHS) during Physical Exercise under Extreme Weather Conditions

EHS can be due to the production of metabolic heat (internal heat) and/or an overload of environmental heat (external heat), and occurs when the thermoregulatory system is disabled due to excessive heat production or inhibition of heat loss (i.e., a decreased sweating response) [45,61] EHS occurs in endurance sport because of the individual overrides the all-important behavioral protection against heat storage—self-pacing, that is, the reduction of work rate in response to perceived heat stress.

EHS can occur in various environmental conditions, but it is most commonly associated with long periods of extreme heat or solar stress alongside exposure to high humidity [29]. The diagnostic signs of EHS are collapse, aggressiveness and irritability, confusion, convulsions, and alterations of consciousness [61], and it can even lead to death in some cases [52]. It has an estimated incidence of 1 in every 1000 athletes [62], and it is frequently observed in adventure races, climbing, hiking, cycling competitions, and ultra-endurance races [52,62]. In the latter, it is most common in marathons [14,44,45] and in ultra-marathons several cases of athletes that did not finish because of heat-related factors, as an inadequately heat acclimatization have been reported [29,35].

Based on the analysis of the different studies found, inadequate acclimatization and the DH of athletes can be suggested as the main causes of this adverse outcome. These data suggest that a fundamental part of the avoidance of DH is an adequate pre-competition preparation, which should include: the planning of rest and hydration depending on the conditions in which the competition takes place, the performance of a pre-acclimatization protocol, and the development of a plan of emergency action for EHS situations [62]. In addition, a pre-competition evaluation would identify the athletes at risk, providing the opportunity to carry out a nutritional and water-intake intervention that supplies the athletes with the necessary information for a safe participation in the sporting event.

### 4.4. Hypothermia (HT)

Reports of deaths related to swimming among participants in endurance and ultra-endurance competitions, such as triathlon and open-water swimming, have generated a climate of concern among organizers, participants, and those athletes who consider enrolling in such events. The progressive increase in the number of participants in these events has also resulted in an increase in the number of injuries and sudden deaths. It is suspected that the majority of deaths in triathlon competitions occur during the swimming stage [63].

In cold waters, the rectal temperature can continue to fall even after the end of the swimming stage; this, together with the relative humidity that the athletes experience when subsequently continuing with the triathlon bike stage, can trigger a greater fall in body temperature and a deterioration in the performance of the athletes [64]. According to the data we have obtained, the average incidence of HT is 1.7% for all studies included and approximately 1.5% among athletes who participate in open-water swimming events [27,50,51].

Based on the analysis of the different studies included in this review, HT does not appear to be a mechanism that triggers death in athletes. The available evidence is limited, but cardiac arrhythmias appear to be the most likely cause of death related to swimming events. Although the mechanisms of cardiac arrhythmias are not clearly defined, there is a theoretical relationship between body water balance and their development [63].

The incidence of HT is lower than those of other adverse outcomes, but it is not limited to sports involving swimming races, it also occurs in other sports such as mountaineering and mountain races, in which high altitude causes a descent in temperature and increases the probability that athletes suffer this condition [27]. It has been reported that HT, as a non-traumatic medical pathology related to the environment, accounts for 68.9% of the rescues of mountain athletes [56].

To avoid and prevent most of these problems that endanger the health of athletes, it is necessary to continue developing and discussing action plans in case of emergency, to make correct diagnoses, and to inform athletes of the importance of covering their water and electrolyte needs during competitions. These suggestions are reasonable alterations that can improve the safety of these sporting events and minimize the risks to which the athletes are exposed. At nutritional level because in cold weather the active body still sweats and needs fluids, and warm fluids or at room temperature can help to protect against hypothermia, so good strategies of fluid management before, during, and after exercise could contribute to prevent hypothermia [20].

## 5. Conclusions

The main conclusions of the present study are: (1) The adverse outcomes with the highest incidence in endurance and ultra-endurance sports is DH, followed by EAH, GI, and HT problems; (2) these conditions affect not only performance, but also the health of participants, and symptoms of their occurrence should be a signal for affected athletes to pull out of the event; (3) given the serious consequences of the development of EAH, the need to improve the dissemination of nutritional information and education for endurance and ultra-endurance athletes—based on individualized strategies for the consumption of liquids and electrolytes—should be highlighted, to minimize the risk of developing EAH; (4) DH is a predisposing factor in the development of EAH. Athletes participating in endurance and ultra-endurance competitions should keep in mind that both prior acclimatization to the weather conditions of the competition and an adequate hydro-electrolyte balance reduce the risk of DH and therefore the risk of suffering from EAH; (5) the introduction in the diet of athletes of products or supplements that they have not consumed before is discouraged, because the side-effects that their consumption can trigger are unknown; (6) to prevent athletes from suffering from EHS, they must undergo a previous protocol of acclimatization to the conditions in which the competition will take place; and (7) despite having a lower incidence, it is important to pay attention to the body temperature of athletes who participate in swimming events or in cold-weather conditions, thus avoiding the development of HT.

## Figures and Tables

**Table 1:** Nutrition-related adverse outcomes in endurance and ultra-endurance competitions and sports, in cohort studies.

Study	Objectives	Sample	Sporting Competition	Adverse Outcomes	Incidence	Causes	Conclusions
Parkkali et al. (2017) [24]	-To determine the origin and prevalence of GI problems in participants in triathlon y duathlon -To adopt preventative measures for future events.	239 athletes -80 F -159 M	Triathlon and duathlon, The Netherlands (2015)	Acute GI illnesses	GI illnesses: 73 cases (30.5%) -Stomach pain: 52 cases (71%) -Nausea: 56 cases (77%) -Diarrhea: 43 cases (59%) -Vomiting: 28 cases (38%)	Consumption of energy drinks and ingestion of sips of water from the canal in which the swimming event took place.	-The rate of appearance of the symptoms in the 2 days after the event suggested a viral origin, which was confirmed by the detection of norovirus in feces samples. -The open-water swimming events were the most probable source of the infection. -No food or drink, except the energy drinks, was associated with the GI illnesses.
Meyer et al. (2017) [25]	-To evaluate the incidence of diarrhea in a hiking trail. -To determine the seriousness and causes of this GI problem.	737 athletes -281 F -420 M -36 unspecified	John Muir Trail (JMT), long-distance hiking trail, USA (2014)	GI problems: diarrhea.	Diarrhea: 121 cases (16.4%) -Mild: 99 cases (82%) -Moderate: 16 cases (13%) -Severe: 6 cases (5%)	Fecal contamination of the natural sources of water ingested, together with poor handwashing on the trail.	-The incidence of diarrhea is relatively low compared with other long-distance trails in the USA. -By following standard hygiene recommendations and using filtration or treatment methods for the water, the prevalence of GI problems in this type of event could be reduced.
Magee et al. (2017) [26]	To evaluate the hydration and physical state of university athletes according to the sports and disciplines practiced.	429 athletes Runners: -54 M Cyclists: -7 F -22 M	-Runners: 5 cycles of 2 km on uneven terrain. -Cyclists: cycles of 100 km with a rise in altitude of 1000 m. Ireland (2016)	DH	Total DH: 187 cases (43.6%) -Runners, post-training: 6 cases (12%) -Cyclists, post-training: 13 cases (45%)	Lack of knowledge about the liquid requirements and nutritional values of the athletes.	-Many athletes consume insufficient liquid during physical exercise. -The opportunities to drink water and other liquids during the events are too limited. -There are more drinks-breaks during team sports; even so, the participants do not consume enough water to avoid DH.
Brustia et al. (2016) [27]	-To obtain information about the risks involved in mountain sports, so that adequate information can be supplied. -To develop recommendations for health and safety teams.	202 athletes	Endurance sports in the French Alps, at 2500 m altitude. (2013)	Injuries due to cold and HT.	Injuries due to cold: 13 cases (6.4%) Due to HT: 9 cases (4.4%)	Exposure to high altitudes for long periods of time, in addition to the use of thermal protection inferior to that used by climbers in winter.	-The medical risks and trauma cases produced at high altitude affected 1% of the participants. -Head injuries and traumas are the most frequent types at high altitude. -Altitude sickness and cold-related injuries are more frequent in summer, during climbing activities.
Chlíbková et al. (2016) [28]	To determine the state of hydration before and after a competition and the incidence of EAH in ultra-endurance athletes.	113 athletes -25 F -88 M	Ultra-endurance in the Czech Republic. (2012–2013) -4 cycle races of 24 h in the mountains. -3 foot races: 2 of 24 h and 1 of 100 km	DH and EAH.	EAH: 13 cases (11.5%) DH: 30 cases (27%)	Loss of sodium in the sweat during the races. Over-ingestion of drinks and greater retention of liquids.	-Incidence of EAH greater than that reported previously in ultra-endurance athletes. -Hyper-hydration before the race did not protect the athletes. -The results obtained indicate that the concentration of sodium [Na^+^] in the blood is still the only viable way of determining EAH in endurance athletes.
Danz et al. (2016) [13]	To evaluate the rates of EAH in an Ironman competition.	1089 athletes -157 F -932 M	Ironman championships held in Europe (2005–2013)	EAH.	EAH: 115 cases (10.6%) -Mild: 95 cases (8.7%) -Severe: 17 cases (1.6%) -Critical: 3 cases (0.3%)	Association between: -EAH and F -EAH and those that took longer to complete the events.	-EAH has a high incidence among Ironman competitors. -F competitors and those that took ≥9 h seem to be more susceptible to EAH.
Joslin et al. (2016) [29]	To determine the number of medical complications related to EHS and the number of competitors who, for this reason, did not complete the race.	326 athletes	Ultra-marathons of various stages, in the Amazonian jungle, Brazil (2008–2013)	EHS.	Heat related problems: 29 cases (8.9%)	Incorrect acclimatization before the event and inappropriate rest between stages.	Obligatory rests during ultra-endurance competitions in tropical conditions are probably the best way to improve the safety of the athletes and their acclimatization to the heat.
Stuempfle et al. (2015) [30]	To examine the incidence, the seriousness, and the time of appearance of symptoms of GI problems in athletes who complete the event, and in those who do not.	272 athletes -56 F -216 M	Ultra-marathon of 161 km, USA. (2013)	GI illnesses.	GI symptoms: 261 cases (96%) -Nausea: 157 cases (60.3%) -Vomiting: 92 cases (35.4%) -Stomach cramps: 83 cases (31.9%) -Intestinal cramps: 63 cases (24.1%) -Diarrhea: 58 cases (22.2%) -GI hemorrhages: 4 cases (1.5%)	Multifactorial origin, including an altered physiology, mechanical factors, and the intake of water and food before the competition.	-GI symptoms were experienced by most of the runners (96.0%). -The results obtained seem to confirm that GI symptoms are very common during ultra-marathons; in particular, nausea, a key problem that can determine whether or not a competitor can complete the event.
Cairns et al. (2015) [12]	-To examine the incidence of EAH during and after an ultra-marathon. -To evaluate the non-osmotic stimuli with distinct concentrations of arginine and vasopressin in runners suffering hyponatremia.	15 athletes -3 F -12 M	Ultra-endurance, Great North Walk (GNW) 100s, Australia. (2013)	EAH.	EAH: 10 cases (66.7%)	High consumption of alcohol, rise in body weight during the event, low body weight, female sex, slow pace, lack of experience in these competitions.	-EAH incidence of 67% in endurance athletes, which highlights the need to improve the information and education, to minimize the risk of EAH. -The incidence of EAH was greater in F than in M.
Hoffman et al. (2013) [31]	-To analyze cases of EAH. -To define the relationship between [Na^+^] and changes in body eight. -To examine the interactions between the incidence of EAH, ambient temperature, and hydration state.	887 athletes	WSER ultra-marathon, 161 km, USA. (2009–2012)	DH and EAH.	DH: 164 cases (18.5%) EAH: 101 cases (15.1%)	The ambient temperature and an inverse relationship between [Na^+^] and the percentage change in body weight.	-The incidence of EAH may be higher in ultra-marathons of 161 km, especially at higher temperatures—when the over-hydration is lower and DH is greater. -Although weight loss does not seem to have an adverse effect on performance, ultra-endurance athletes should not take excessive supplementary Na or drink too much liquid.
Rüst et al. (2012) [32]	To investigate the prevalence of EAH in masculine triathletes competing in the Triple Iron triathlon.	45 M	“Triple Iron” Triathlon, Germany. (2007)	EAH.	EAH: 8 cases (17.8%)	Excessive intake of liquids, more inappropriate secretion of ADH and complications in the mobilization of Na+.	-The prevalence of EAH in this event is greater, based on existing reports on Ironman athletes. -In comparison with marathon runners, the athletes studied here, who compete for >24 h, seem to have a greater risk of developing EAH.
Mexia et al. (2013) [33]	-To evaluate risk factors associated with GI problems. -To determine the use of preventative measures. -To compare the findings with those of previous years regarding GI problems and evaluate risk and protective factors.	11721 athletes	“Birkebeinerrittet” mountain bike race, Norway. (2010)	GI illnesses.	Diarrhea: 572 cases (4.9%)	Exposure to mud in the face or mouth during the competition. The absence of a mudguard increased the risk.	-Exposure to mud with fecal contamination is a risk factor. The environmental measures that could reduce this exposure include the elimination of grazing livestock from close to the track and the reduction in the size of muddy areas. -Improvement of preventative strategies.
Hoffman et al. (2012) [34]	-To determine the incidence of EAH, the associated biochemical parameters, and risk factors. -To check if there is an association between the [Na^+^] in the blood after the race and the changes in body mass of the participants.	47 athletes	Ultramarathon WSER de 161 Km, EEUU. (2009)	EAH and associated nausea	EAH: 14 cases (30%) Nausea: 22 cases (46.8%)	It is associated with DH, since it suggests that the loss of sodium can be a factor in the development of EAH during events of long duration at high temperatures.	-EAH may be a common condition (incidence of 30%) among the finalists of an ultra-marathon. -It is not unusual for athletes with EAH to suffer DH. -The development of EAH was not related to age, sex or the time taken to complete the event, but the athletes with EAH had participated in fewer ultra- marathons.
Hoffman et al. (2011) [35]	To explore demographic characteristics and problems that affect the sport performance during an ultra-marathon.	489 athletes	WSER ultra-marathon (161 km) and Vermont 100, North America. (2009)	Medicinal- nutritional problems that prevented 232 participants from completing the event	Nausea and/or vomiting: 92 cases (39.6%) DH: 11 cases (4.7%) Inadequately heat acclimatization: 65 cases (28.1%)	The ambient temperature, the amount of training, or experience in successfully completing this type of event.	-More than half of the ultra-marathon runners suffered a problem that prevented them from finishing the competition and led to an average of 21 days of recovery and preparation for later races. -Nausea and/or vomiting are an important problem that limits the performance of participants in ultra-marathons. -The athletes affected by nausea and/or vomiting took longer to complete the race than those not suffering these symptoms.
Bruso et al. (2010) [36]	To explore risk factors associated with development of EAH and rhabdomyolysis during an ultra-marathon.	400 athletes	WSER ultra-marathon (161 km), USA. (2009)	EAH.	EAH: 5 cases (1.25%)	The causes of the appearance of EAH in this competition have not been established.	-One consistent characteristic was that all 5 individuals affected received a normal saline solution, intravenously, as part of their initial treatment. -Extreme precaution is recommended in the use of normal saline solution, given intravenously, especially if there are symptoms of cerebral encephalopathy, which could be worsened by this action.
Griffiths et al. (2010) [37]	To investigate the causes of an outbreak of diarrhea during a mountain bike competition.	347 athletes -45 F -299 M -3 unspecified	Mountain bike competition, UK. (2008)	GI illnesses.	GI illnesses: 161 cases (46.5%) -Diarrhea: 151 cases (43.5%) -Abdominal pain: 131 cases (37.7%) -Nausea: 91 cases (26.2%) -Vomiting: 31 cases (8.9%) -Blood in feces: 15 cases (4.3%)	Outbreak caused by Campylobacter and spread by the accidental ingestion of mud, as well as by the use of water contaminated in the same way to fill the water bottles.	-The successful use of the Internet, to carry out a quick and efficient investigation into an outbreak of diarrhea and other symptoms associated with a mountain bike competition, was demonstrated. -The microbiological risks are an inherent part of this type of sport and the hygiene measures must be very strict to minimize the risk of infection.
Kipps et al. (2011) [38]	To determine the incidence of EAH in the runners who participate in a marathon.	88 athletes	London marathon, United Kingdom. (2003)	EAH.	EAH: 11 cases (12.5%)	Some runners probably started the race in a state of hyperhydration.	-A significant proportion (12.5%) of the participants developed asymptomatic EAH after completing the race. Although they consumed more fluids, the ingestion of fluids was not related to the increase in body weight. -4 of the 11 runners with hyponatremia lost weight during the race, reinforcing the idea of an additional factor in the development of EAH.
Chlíbková et al. (2015) [39]	-Identification of athletes that develop EAH and rhabdomyolysis simultaneously. -To study hyponatremic and normonatremic athletes and try to find biochemical factors common to both EAH and rhabdomyolysis.	145 athletes	7 ultra-endurance races in the Czech Republic (2012–2013): -5 cycle races (24 h, mountain and trilogy) -2 foot races (24 h and 100 km)	EAH (113 completed the competition)	EAH: 13 cases (11.5%)	Secretion of arginine and vasopressin (antidiuretic hormone), and loss of liquid from muscle.	-Rhabdomyolysis induced by the exercise was more frequent in hyponatremic athletes than in normonatremic athletes. -The incidence of rhabdomyolysis tended to be greater among ultra-runners than among mountain cyclists. -The mechanism that could explain the relationship between rhabdomyolysis and EAH was not determined.

M = male; F = female; GI: gastrointestinal; EAH: exercise-associated hyponatremia; EHS: heat stroke by exertion; DH: dehydration; HT: hypothermia.

**Table 2:** Nutrition-related adverse outcomes in competitions and endurance and ultra-endurance sports, investigated in randomized clinical trials.

Study	Objectives	Sample	Sporting Competition	Adverse Outcomes	Incidence	Causes	Conclusions
Armstrong et al. (2017) [40]	-To evaluate the state of cyclists who began the competition with normal plasma [Na^+^], but finished it with 130 mmol/L. -Comparison of the values of these cyclists with those of a control group of 31 normonatremic cyclists.	33 M	Open-air cycling competition, “Hotter’N Hell Hundred” (164 km), USA. (2016)	EAH.	EAH: 2 cases (6%)	Recommendations about the total intake of liquids and the change in the concentration of [Na^+^] determined the development of this condition.	-Each athlete must develop their own personalized plan for water intake, which includes measures during training, such as the calculation of the rate of sweating. -The precision of this method depends on the coincidence in the field simulation, regarding the environmental conditions and the intensity of the exercise.
Cutrufello et al. (2016) [41]	-To examine the relationship between the density of the urine, the bioelectrical impedance, and the body mass, before and after a marathon. -To check the hypothesis regarding the loss of body mass and of total body water that marathon runners experience.	35 athletes -10 F -25 M	Marathon in the USA (2015)	Hydration state.	Hyperhydration: 22 cases (62.8%)	Lack of knowledge about hydration in this type of competition.	-The body mass values seem to reflect changes in hydration, but not when using the values of a single athlete. -The hydration regime before and after a marathon should be considered in future investigations, and the time of completion of the race should be compared with the bioelectrical impedance results.
Valentino et al. (2016) [42]	-To determine if DH (defined as body mass losses ≥3%) leads to critical changes in body temperature during an ultra-marathon of 161 km. -To check if the hydration state directly affects the body temperature.	30 athletes -7 F -23 M	WSER ultra-marathon (161 km), USA (2014)	Hydration state (Only 20 athletes managed to complete the race)	DH: 10 cases (50%) GI illnesses: 5 cases (16.7%)	The athletes who lost a higher percentage of body mass completed the race more quickly.	-The losses of body mass (3–4%) were not related to the state of HT. -To avoid losses of body mass ≥2% during such events, it is not necessary to achieve a state of hyperhydration since it does not prevent HT.
Knechtle et al. (2011) [43]	-To investigate the prevalence of EAH in athletes in ultra-endurance sports modalities. -Comparison of the prevalence of EAH in these athletes with that in marathon runners and Ironman triathletes.	200 athletes	Ultra-endurance races in Switzerland (2007–2009): -Swimming marathon in open water -2 cycling marathons -2 running marathons, 1 race of 100 km	EAH.	EAH: 12 cases (6%)	Consumption of alcohol and a high frequency of intake of liquid.	-The mean prevalence of EAH among the athletes studied was 6%. -The prevalence of EAH was higher than that found elsewhere for marathon, ultra-marathon, and Ironman competitors.

M = male; F = female; GI: gastrointestinal; EAH: exercise-associated hyponatremia; EHS: heat stroke by exertion; DH: dehydration; HT: hypothermia.

**Table 3:** Nutrition-related adverse outcomes in competitions and endurance and ultra-endurance sports, investigated in case studies.

Study	Objectives	Sample	Sporting Competition	Adverse Outcomes	Incidence	Causes	Conclusions
Gomm et al. (2016) [44]	To demonstrate the effectiveness and safety of a portable medical device for chilling that acts on the endothermic surface.	3 M	2 marathons, UK. (2015)	EHS and other, related problems.	EHS: 3 cases (100%) Vomiting: 1 case (33.3%)	Environmental conditions during the competitions.	-The basis of the treatment is rapid attention and cooling; the fastest cooling rates were with water and ice baths. This reduced complications, being associated with a better long-term prognosis.
Six et al. (2016) [8]	To identify the source of infection and document the reach of an outbreak of acute gastroenteritis that occurred during an obstacle adventure race.	729 athletes -332 F -397 M	Obstacle adventure race (13 km, 22 obstacles), France. (2015)	GI illnesses.	Acute gastroenteritis: 375 cases (50.3%)	Person to person transmission of a norovirus, the source of which was contaminated sewage sludge.	-A series of recommendations should be proposed to reduce the risk of infection among athletes in this type of event. -The runners in and organizers of these events should be aware of the possible risks involved in accidental ingestion of dirty water, avoiding areas contaminated by animal feces.
Carvalho et al. (2016) [45]	Identification, diagnosis, and evolution of an athlete who suffered from EHS together with other medical complications during a marathon.	1 M	Marathon (the subject only completed 16 km), Portugal. (2016)	EHS and related complications.	EHS: 1 case (100%) Hypoglycemia: 1 case (100%)	Climatic changes and certain non-prescribed medicines could have been the causes.	-The athlete was cooled down in the hospital emergency department after only a few hours of the EHS, which may have contributed to the good outcome and recovery. -This works shows the importance of identifying risk factors, such as intake of medications that affect heat dissipation (antihistamines, anticholinergics, or calcium antagonists), sleep deprivation, and DH.
Smith et al. (2016) [14]	Analysis of the case of monozygotic twins that collapsed after EHS and during the same event, in relatively cold weather.	2 M Monozygotic twins	35-km race, UK. (2016)	EHS.	EHS: 2 cases (100%)	Causes unknown; the subjects denied sleep deprivation and felt hydrated before the start of the race.	-The development of EHS is multifactorial; however, the combination of the responsibilities of the pace of the race, the additional resistance of the backpack weight, and a genetic predisposition are likely to have played an important role.
Roberts et al. (2016) [46]	-Analysis of the case of an athlete who collapsed in two different marathons due to EHS and did not finish either races. -To study a simulation of the return to physical activity, to avoid repeating the circumstances of previous competitions.	1 M	Two marathons, 6 weeks apart. The athlete dropped out of both after 20 km. USA. (2009)	EHS.	EHS: 1 case (100%)	Genetic causes of the EHS were discounted and an inadequate heat tolerance during these competitions was considered as the possible cause.	-The challenge of achieving an adequate hydro-electrolytic balance increases the risk of suffering from EHS during these competitions. -A quick response of spectators and colleagues, to ask for medical help, is crucial to reduce the risks associated with EHS. -To ensure a successful completion of these events, adequate heat tolerance should be guaranteed, as runners may be susceptible to EHS in cold conditions.
Myers et al. (2015) [47]	-To demonstrate the diagnostic challenges and the importance of proper management in cases of EAH. -To confirm that treatment with high volumes of an isotonic solution may delay recovery and may even lead to death.	1 F	Hiking in the Grand Canyon National Park, approximately 10 km, USA (2008)	EAH and other, related problems.	EAH: 1 case (100%) -Vomiting: 1 case (100%)	Overhydration combined with fluid retention caused by the secretion of arginine-vasopressin.	-The first symptoms of EAH include nausea, vomiting, and headache, which progress rapidly to confusion, altered mental status, seizures, and death if not treated in time. -The recognition of EAH and its treatment with hypertonic saline solution is a safe and effective option. In addition, for sporting events that take place in areas with a desert climate, it would be beneficial to have sodium detection tests and to be able to administer hypertonic saline solutions
Severac et al. (2014) [48]	-To analyze the case of an athlete that, after completing an Ironman event, went to the emergency department with a headache, nausea, and confusion. -To study the causes that triggered the EAH.	1 F	Ironman, France (2012)	EAH and other, related problems.	EAH: 1 case (100%) -Nausea: 1 case (100%)	-Excess fluid consumption compared to the losses of body fluids.	-EAH remains a complication with few diagnoses in endurance sports. In more severe cases, a correct and timely treatment allows complete neurological recovery. -Risk factors have been identified, such as: female sex, use of anti-inflammatories and diuretics, excessively cold or hot weather, the duration and intensity of the event, and excessive fluid intake
Hostler et al. (2014) [49]	To analyze the case of a half-marathon athlete that suffered EHS during a race in relatively mild conditions and compare it with cases of other runners who also suffered EHS in similar climatic conditions.	1 M	Half-marathon, USA. (2012)	EHS.	EHS: 1 case (100%)	-The climatic conditions in an urban stretch of direct sunlight near the finish line.	-EHS requires active cooling therapy but this is rare during a half-marathon; however, EHS can occur even in relatively mild conditions. -The climatic conditions must be directly monitored at multiple points throughout the race, since it is possible for the temperature to be estimated in a single place that is not representative of the heat load on the participants
Bhangu et al. (2010) [50]	-To evaluate if body temperature contributed to the abandonment of an event by competitors. -To evaluate the agreement between the measurements made using oral and tympanic thermometers.	4700 athletes	“Adventure raid”: an open-air event incorporating climbing, swimming, rafting. United Kingdom. (2009)	HT.	HT: 64 cases (1.4%)	-The climatic conditions and the swimming stages contributed to a decline in body temperature of the participants.	-The diagnosis of the doctors did not always coincide with the data obtained using the thermometers, regarding the existence of hypothermia or not in the runners. -The values of the tympanic and oral thermometers had little agreement.
Castro et al. (2009) [51]	To address the risks of HT and hypoglycemia during an open-water swimming competition, to alert doctors about the potential dangers of this type of competition	12 athletes -5 F -7 M	Official open-water swimming competition (10 km), Brazil. (2008)	HT and hypoglycemia.	HT: 10 cases (83%) Hypoglycemia: 0 cases (all but one of the athletes received several doses of maltodextrin)	-Exposure to water for a prolonged period (at least two hours), contributing to the reduction of body temperature.	-HT is a common phenomenon, even in swimmers competing in relatively warm water. Therefore, it could be an important medical concern in such events. -The measurement of body temperature should be a key factor during competitions with these characteristics.
Rae et al. (2008) [52]	To determine why only 4 of the 35,627 athletes competing in cycling races were hospitalized by EHS, and whether the exercise alone could have raised their body temperature enough to cause EHS.	4 M	3 cyclists that participated in races, completing 80–109 km, died shortly after, South Africa. (2002) Another athlete, who completed 56 km in the “Two Oceans” marathon, South Africa. (2006)	EHS and other, related problems.	EHS: 4 cases (100%) -Vomiting: 2 cases (50%) -EAH: 1 case (25%)	-High rates of heat production in unfavorable environmental conditions caused a progressive build-up of body heat.	-The HT of the cases studied may have been the result of failure in the heat-loss mechanisms. -These cases could have been due to excessive endothermia, triggered by physical exertion. A correct diagnosis of excessive endothermia in cases of heat stroke in mild to moderate environmental conditions and the immediate initiation of cooling in all cases of EHS are crucial.

F = female; M = male; GI: gastrointestinal; EAH: exercise-associated hyponatremia; EHS: heat stroke by exertion; DH: dehydration; HT: hypothermia.

**Table 4:** Incidence of adverse outcomes in endurance/ultra-endurance sports in cohort studies.

Incidence (%)				
**DH**	**EAH**	**GI**	**HT**	**EHS**
392/1918 (20.4%)	290/2829 (10.3%)	1302/13,852 (9.4%)	13/202 (6.4%)	n.r.

GI: gastrointestinal; EAH: exercise-associated hyponatremia; EHS: heat stroke by exertion; DH: dehydration; HT: hypothermia; n.r.: not reported.

## Appendix A

Words and search strategy:

(Gastrointestinal problems[Title/Abstract] OR “Dizziness”[Mesh] OR Dizzyness[Title/Abstract] OR Orthostasis[Title/Abstract] OR Lightheadedness[Title/Abstract] OR Light-Headedness[Title/Abstract] OR Light Headedness[Title/Abstract] OR “Nausea”[Mesh] OR Stomach cramps[Title/Abstract] OR Intestinal cramps[Title/Abstract] OR Gastrointestinal cramps[Title/Abstract] OR “Gastrointestinal Hemorrhage”[Mesh] OR Hemorrhage, Gastrointestinal[Title/Abstract] OR Gastrointestinal Hemorrhages[Title/Abstract] OR Hematochezia[Title/Abstract] OR Hematochezias[Title/Abstract] OR “Labor Pain”[Mesh] OR Pain, Labor[Title/Abstract] OR Obstetric Pain [Title/Abstract] OR Pain, Obstetric[Title/Abstract] OR “Vomiting”[Mesh] OR Emesis[Title/Abstract] OR “Diarrhea”[Mesh] OR Diarrheas[Title/Abstract] OR “Hyponatremia”[Mesh] OR Hyponatremias[Title/Abstract] OR “Hypoglycemia”[Mesh] OR Postprandial Hypoglycemia[Title/Abstract] OR Hypoglycemia, Postprandial[Title/Abstract] OR Reactive Hypoglycemia[Title/Abstract] OR Hypoglycemia, Reactive[Title/Abstract] OR Fasting Hypoglycemia[Title/Abstract] OR Hypoglycemia, Fasting[Title/Abstract] OR Postabsorptive Hypoglycemia[Title/Abstract] OR Hypoglycemia, Postabsorptive[Title/Abstract] OR “Dehydration”[Mesh] OR Water Stress[Title/Abstract] OR Stress, Water[Title/Abstract] OR “Heat Stroke”[Mesh] OR Heat Strokes[Title/Abstract] OR Stroke, Heat[Title/Abstract] OR Strokes, Heat[Title/Abstract] OR Heatstroke[Title/Abstract] OR Heatstrokes[Title/Abstract] OR “Hypothermia”[Mesh] OR Hypothermias[Title/Abstract] OR Hypothermia, Accidental[Title/Abstract] OR Accidental Hypothermia[Title/Abstract] OR Accidental Hypothermias[Title/Abstract] OR Hypothermias, Accidental[Title/Abstract] OR “Fever”[Mesh] OR Fevers[Title/Abstract] OR Pyrexia[Title/Abstract] OR Pyrexias[Title/Abstract] OR Hyperthermia[Title/Abstract] OR Hyperthermias[Title/Abstract] OR “Endotoxemia”[Mesh] OR Endotoxemias[Title/Abstract]) AND (“Sports”[Mesh] OR Sport[Title/Abstract] OR Athletics[Title/Abstract] OR Athletic[Title/Abstract] OR Endurance Sports[Title/Abstract] OR Endurance Exercise[Title/Abstract] OR Endurance Events[Title/Abstract] OR Endurance Athletes[Title/Abstract] OR “Bicycling”[Mesh] OR Bicycling[Title/Abstract] OR Mountain bike[Title/Abstract] OR “Mountaineering”[Mesh] OR Mountaineerings[Title/Abstract] OR “Running “[Mesh] OR Runnings[Title/Abstract] OR “Jogging”[Mesh] OR Joggings[Title/Abstract] OR Marathon[Title/Abstract] OR Marathon runner[Title/Abstract] OR Marathon runners[Title/Abstract] OR Runner[Title/Abstract] OR Runners[Title/Abstract] OR Race runners[Title/Abstract] OR Foot race[Title/Abstract] OR Ultra-Marathon Runner[Title/Abstract] OR Ultra-Marathon Runners[Title/Abstract] OR Ultramarathon Runner[Title/Abstract] OR Ultramarathon Runners[Title/Abstract] OR Triathlon[Title/Abstract]).
